# Treatment of Severe Periodontitis using Exosome-Mediated Combination Therapies: A Retrospective Cohort Study

**DOI:** 10.3290/j.ohpd.c_2745

**Published:** 2026-06-30

**Authors:** Scott Froum, Nathan E. Estrin, Hyun Jae Cho, Nima Farshidfar, Paras Ahmad, Richard J. Miron

**Affiliations:** a Scott Froum Professor, Department of Periodontology, School of Dental Medicine, Stony Brook University, Stony Brook, NY, USA. Conceptualization, methodology, formal analysis, investigation, resources, wrote original draft, reviewed and edited the manuscript, supervision, project administration.; b Nathan E. Estrin Professor, The University of Iowa College of Dentistry and Dental Clinics, Iowa City, IA, USA. Conceptualization, methodology, formal analysis, investigation, resources, wrote original draft, reviewed and edited the manuscript, supervision, supervision, project administration.; c Hyun Jae Cho Periodontist, Department of Periodontology, School of Dental Medicine, Stony Brook University, Stony Brook, NY, USA. Methodology, formal analysis, investigation, resources, wrote original draft, reviewed and edited the manuscript, project administration.; d Nima Farshidfar PhD Student, Department of Periodontology, University of Bern, Bern, Switzerland. Methodology, formal analysis, resources, reviewed and edited the manuscript, project administration.; e Paras Ahmad Post-doctoral Student, Department of Research, The Miron Lab, Florida, USA. Methodology, formal analysis, resources, wrote original draft, reviewed and edited the manuscript, project administration.; f Richard J. Miron Professor, Department of Periodontology, University of Bern, Bern, Switzerland; Department of Research, The Miron Lab, Jupiter, FL, USA. Conceptualization, methodology, formal analysis, investigation, resources, wrote original draft, reviewed and edited the manuscript, supervision, project administration. All authors read and agreed to the published version of the manuscript.

**Keywords:** bone regeneration, extracellular vesicles, exosomes, periodontitis, tissue scaffolds.

## Abstract

**Purpose:**

This pilot retrospective case series aimed to evaluate the 6-month clinical outcomes and safety of a combined regenerative protocol utilizing exosomes specifically designed for periodontal regeneration (Periosomes), with 90% anorganic bovine bone/10% collagen (ABBMC), and horizontal platelet-rich fibrin (H-PRF) for the treatment of advanced periodontal osseous defects.

**Materials and Methods:**

This study analyzed stage-III (severe) periodontitis patients (from poor to hopeless prognosis) who underwent periodontal surgery using Periosomes with an ABBC/H-PRF scaffold with a 6-month follow-up. Complete periodontal charting, including probing depth (PD), gingival margin (GM), bleeding on probing (BOP), clinical attachment loss (CAL), gingival index (GI), plaque index (PI), and tooth mobility, was assessed at baseline and the follow-up. The study included 13 patients (8 females and 5 males) aged 29 to 73 years, with 23 periodontal defects of one-walled (60.9%) and two-walled (39.1%) defect morphology.

**Results:**

The sites treated with Periosomes showed statistically significant reductions in PD from baseline to six months in one-walled defects (8.50 ± 2.41 mm to 3.14 ± 0.77; p < 0.0001) and two-walled defects (7.56 ± 1.13 mm to 3.22 ± 0.44; p < 0.0001) as well as in CAL from 9.14 ± 3.01 mm to 4.79 ± 2.17 (p < 0.0001) in one-walled defects and from 7.22 ± 1.56 mm to 3.56 ± 1.01 (p < 0.0001) in and two-walled defects. Furthermore, frequency distribution analysis found that 96% of sites attained residual PD < 5 mm and 92% showed CAL gains ≥3 mm at the 6-month follow-up.

**Conclusion:**

The combined use of Periosomes, ABBMC, and H-PRF was associated with favorable short-term clinical improvements in advanced periodontal osseous defects with no reported adverse events. To our knowledge, this is the first human clinical study assessing the use of exosomes in periodontal regenerative therapy. However, controlled clinical trials with comparator groups and longer follow-up are necessary to evaluate the independent contribution of exosomes in periodontal regeneration.

Periodontitis is characterized by both host and microbial-mediated inflammation, resulting in the loss of tooth-supporting tissues.^1,6,7,44^ According to data examining the disease’s prevalence from a nationwide study carried out in the US (NHANES), 64.7 million adults, or more than 47% of the population, were affected, with 38.5% of those cases being moderate or severe (stage III or stage IV).^11^ When treating periodontal disease, regeneration of the lost periodontal supportive structures is the ultimate objective.^10^ Periodontal regeneration includes reconstruction of the previously lost cementum, bone, and periodontal ligament, and its success is contingent upon the existence of intrabony defects caused by vertical bone loss, which would contain walls to hold biological agents and biomaterials.^44,46,47^ Various strategies exist for successful periodontal regeneration, including a combination of bone grafting materials, barrier membranes, and biological growth factors.^32,36,38^

The additional use of growth factors has been proposed as a potential method to further enhance periodontal regeneration.^18,29,30^ While stem cell therapy remains relatively rarely utilized in clinical practice owing primarily to safety concerns, recombinant human growth factors such as platelet-derived growth factor (rhPDGF), enamel matrix derivative (EMD), and bone morphogenetic protein-2 (rhBMP2) have been more popular due to their greater safety and availability.^5,16,23,30,33^ Nevertheless, the integration of growth factors in the dental clinic is still contentious. For example, their application is restricted in various countries, e.g., in Europe, and the high cost of certain recombinant proteins restricts their full use, thereby underscoring the necessity for cell-free therapeutic strategies.^19,45^

Regarding tissue regeneration, the potential of exosomes has been empirically evaluated in medicine, with over 5000 publications being published on the topic yearly,^4,15,31^ but has seldom been investigated in clinical dentistry.^18^ Exosomes are a category of extracellular vesicles characterized by a diameter ranging between 50 and 150 nm, which are abundant in RNA, lipids, proteins, and other bioactive molecules.^31^ They function as vectors for the transport of signaling molecules, thereby facilitating intercellular communication.^31^ Exosomes also exhibit significant potential in promoting bone regeneration without eliciting an immune response, since they do not carry MHC-I or -II receptors.^2,20^

In a recent scoping review performed by our group, 113 research articles examined the use of exosomes specifically in regenerative dentistry, with all of these studies displaying positive results.^27^ Specifically in periodontal regeneration, 34 pre-clinical studies were investigated with exosomes utilized from various sources, such as periodontal-ligament stem cells (PDLC-Exos), gingival mesenchymal cells (GMSC-Exos), and dental-pulp stem cells (DPSC-Exos), with the target cell line mainly human periodontal ligament cells.^3,26,27,31,42^ In-vitro studies demonstrated the ability of exosomes to stimulate osteogenesis, migration, and proliferation of various cell types even under lipopolysaccharide-induced inflammatory conditions.^3^ Furthermore, local injections of exosomes within periodontal tissues and/or when mixed with bone grafting materials have reported positive periodontal regeneration in various animal models.^27^ It is noteworthy, however, that despite these animal models showing promising results and their established safety and widespread daily use in other fields of medicine, the only reported clinical publication thus far in dentistry is a case report from our group in which exosomes were utilized for horizontal ridge augmentation.^12^

Therefore, the present retrospective case series sought to evaluate short-term clinical outcomes and safety of a combined regenerative protocol utilizing exosomes (Periosomes) combined with 90% anorganic bovine bone and 10% collagen (ABBC) and horizontal platelet-rich fibrin (H-PRF) for the treatment of advanced periodontal bone defects with poor to hopeless prognosis, with periodontal clinical measurements and radiographic assessments conducted at baseline and 6 months post-operatively.

## MATERIALS AND METHODS

### Ethical Considerations and Patient Selection 

Before data collection, an institutional review board (IRB) exemption was obtained for the retrospective chart analysis from Sterling IRB (ID: 12079-RJMiron). All identified patients were treated by a single clinician (SF). In this retrospective cohort study, two authors (SF and HC) reviewed the medical records of subjects diagnosed with Stage III or Stage IV generalized or localized periodontitis who underwent periodontal surgery with Periosomes at a private office setting in New York.

### Eligibility Criteria

The following inclusion criteria were applied: (a) ≥18 years old non-smokers in good systemic health or with controlled medical conditions (American Society of Anesthesiology [ASA] I or II);^39^ (b) patients who completed non-surgical periodontal therapy (NSPT; without systemic antibiotics) at least three months before the regenerative procedure with a plaque score of less than 30%; (c) presence of one or more intrabony defects (>3mm) treated with reconstructive periodontal surgery using exosomes; and (d) availability of pre-and post-operative dental records. All patients participated in supportive periodontal therapy during the six-month follow-up post-surgery.

The following exclusion criteria were applied: (a) teeth used as abutments for fixed dental prostheses; (b) inadequate endodontic treatment and/or restoration; (c) history of smoking; (d) uncontrolled diabetes or other systemic conditions affecting the periodontal health; (e) missing pre-operative periodontal charts; (f) lack of enrollment or non-compliance with supportive periodontal therapy or plaque score greater than 30%; and (g) follow-up duration of < six months.

### Interventions

Informed consent was provided prior to drawing blood and use of exosomes to conduct the outlined experiments.

All subjects received treatment according to a comprehensive periodontal treatment plan. Initial oral hygiene instructions were provided, followed by NSPT without the use of systemic antibiotics. After approximately six months of supportive periodontal therapy, a periodontal examination was conducted for each patient, including comprehensive periodontal charting. At the re-evaluation, patients with teeth showing persistent periodontal pockets of ≥ 6 mm were subsequently scheduled for periodontal reconstructive surgery using exosomes (Periosomes; proprietary exosomes prepared under Good Manufacturing Practices [GMP] standards derived from placental tissue at NeoBiosis, Gainesville, FL, USA), horizontal platelet-rich fibrin (H-PRF) using a 700 RCF (relative centrifugal force) for an 8-min protocol in glass solid PRF tubes (Bio-PRF; Venice, FL, USA) and ABBC scaffold (Bio-Oss Collagen; Geistlich, Switzerland).

All patients underwent a standardized surgical and post-surgical protocol. 0.5 ml (80 billion parts per million) of the Periosomes (NeoBiosis; Gainesville, FL, USA) were defrosted at room temperature for 15 min and then combined with 250 mg of ABBC for an additional 15 min. Local anesthesia was administered using articaine HCl 4% with epinephrine 1:200,000. All teeth with class II and class III mobility were splinted with an intracoronal splint. Sulcular incisions around the defects were made with a 15C blade, followed by a reflection of a full-thickness to split-thickness (at the mucogingival junction) mucoperiosteal flap, allowing both access to the defect and flap advancement for primary closure during healing. Debridement was performed using surgical round diamond burs to remove granulation tissue, followed by manual debridement and decontamination of the defect and the inner flap layer with a 9.3-µm carbon dioxide laser (SOLEA; Waltham, MA, USA) set at high power, 60% cutting speed, 13.9 W, 25% water, and 1.0 mm spot size. After curettage and root planing, root surfaces were detoxified with 24% EDTA Straumann Prefgel (Straumann; Basel, Switzerland) for 2 min and thoroughly rinsed with ozonated sterile saline. The graft complex was applied to the defect with a 20% overfill, and H-PRF membranes were placed over the graft material for containment.^14,25,28^ Flaps were advanced to achieve primary closure with 4-0 Vicryl Ethicon sling sutures. Once hemostasis was established, a peri-acryl dressing was placed, and vestibular release was performed with the laser to relieve tension on the sutured flap when necessary. Postoperative care included amoxicillin 500 mg thrice daily for three days, with analgesics as required, and a homeopathic oral care recovery kit (StellaLife; Aventura, FL, USA) for post-operative recovery.^13^ Patients were scheduled for follow-up visits at 2 weeks (for suture removal), 6 weeks, 12 weeks, and 6 months post-operatively. Oral hygiene instructions were reinforced at each follow-up visit. Clinical parameters recorded at the initial visit (baseline) and 6 months post-surgery included plaque index (PI), gingival index (GI), probing depth (PD), clinical attachment loss (CAL), gingival margin (GM), bleeding on probing (BOP), and tooth mobility.

### Statistical Analysis 

Given the exploratory nature of this pilot case series, a formal calculation of sample size was not performed. The periodontal parameters were manually recorded and entered into the same tabular software by two investigators independently (SF and HJC), utilizing the dental and periodontal records of the patients. Any inconsistencies were rectified following a thorough review of the original documents. The patients’ demographics as well as periodontal measurements were expressed as mean ± SD. Together with mean-based comparisons, a frequency distribution analysis of residual PD at 6 months and CAL gains was conducted. Residual PD scores were classified into clinically relevant cutoffs (< 5 mm and ≥ 5 mm), and CAL gain was classified into < 3 mm and 3 ≥ mm gain.

Statistical analyses were conducted utilizing the SPSS software (version 28.0; IBM, Chicago, IL, USA). The assessment of normality was performed using the Shapiro-Wilk test. Most of the data failed to meet the normality assumptions; consequently, the data were transformed using log transformation to make it closer to a normal distribution. The assessment investigated the correlation between the age of patients and clinical outcomes at baseline and the 6-month follow-up utilizing Pearson’s correlation test. Additionally, to assess the correlation between the patients’ sex and clinical parameter outcomes at baseline and the 6-month follow-up, a non-parametric method was used, i.e., point-biserial correlation. Since sex is categorical (with two levels: male and female), it was converted into a binary variable (0 for male and 1 for female). To assess the influence of treatment on periodontal clinical outcomes at baseline and the 6-month follow-up, a t-test was performed. p-values < 0.05 were regarded as statistically significant.

## RESULTS

### Primary Features of Study Participants

This study included a total of 13 patients with 23 intrabony defects aged between 29 and 73 years (48.38 ± 13.46 years), of which 8 were females and 5 were males. Regarding defect type, one-walled defects were more common, representing 60.9% of cases, while two-walled defects made up 39.1% of defects. Several patients exhibited multiple defect types on various teeth included in the study, with teeth 17, 11, 21, 27, and 41 most frequently being affected. Tooth 17 appeared in three cases, while teeth 11, 21, and 41 each appeared twice. Additionally, defects at the distobuccal site were observed in 11 patients, and the mesiobuccal site was also frequently involved (n=9 patients), indicating a tendency for buccal areas to be more affected (Table 1).

**Table 1:** Primary features of the study participants

Patient number	Sex	Age (years)	Defect type	Tooth No.	Site
1	Female	29	Two-walled	19	Mesiobuccal
2	Female	59	Two-walled Two-walled	14 15	Distobuccal Distobuccal
3	Female	73	One-walled One-walled	2 2	Mesiobuccal Distobuccal
4	Male	51	One-walled One-walled	2 3	Mesiobuccal Distobuccal
5	Male	46	One-walled Two-walled	5 5	Mesiobuccal Distobuccal
6	Female	61	Two-walled Two-walled	10 11	Distobuccal Mesiobuccal
7	Male	45	One-walled One-walled	8 9	Distobuccal Mesiobuccal
8	Female	37	Two-walled Two-walled	8 8	Mesiobuccal Distobuccal
9	Male	29	Two-walled	18	Distobuccal
10	Female	64	One-walled One-walled	8 9	Mesiobuccal Mesiobuccal
11	Female	44	One-walled One-walled	25 25	Mesiobuccal Distobuccal
12	Male	53	One-walled One-walled	2 15	Distobuccal Distobuccal
13	Female	38	One-walled	7	Distobuccal


Two authors (SF and HC) independently assigned each tooth a prognosis according to the McGuire and Nunn classification system.^24^ Any discrepancies between the authors were discussed, and a third author (NE) was included, if necessary, until a prognosis was agreed upon by all authors. All teeth had a poor to hopeless prognosis. Out of the 19 total teeth (23 defects) included in this study, six (32%) were assigned a hopeless prognosis, seven (36%) a questionable prognosis, and six (32%) teeth were assigned a poor prognosis.

### Periodontal Clinical Outcomes

Table 2 displays the mean values of periodontal clinical parameters assessed at baseline and the 6-month follow-up. No adverse events were observed in any of the cases.

**Table 2:** Values of periodontal parameters (mean ± SD) assessed at baseline and 6-month follow-up

Periodontal parameters	Baseline	Six-month follow-up	Mean difference (reduction or gain)	Significance (p-value)
One-walled defect				
Two-walled defects				
Plaque index	2.21 ± 0.70	0.64 ± 0.50	1.57 ± 0.86	p < 0.0001*
Gingival index	2.50 ± 0.52	1.64 ± 1.60	0.86 ± 1.68	p = 0.075
Probing depth (mm)	8.50 ± 2.41	3.14 ± 0.77	5.36 ± 2.53	p < 0.0001*
Clinical attachment level (mm)	9.14 ± 3.01	4.79 ± 2.17	4.35 ± 3.71	p = 0.001*
Gingival margin (mm)	0.64 ± 1.28	1.64 ± 1.60	1.00 ± 2.05	p = 0.08
Tooth mobility	1.14 ± 1.03	0.50 ± 0.52	0.64±1.15	p = 0.05
Plaque index	2.22 ± 0.44	0.56 ± 0.53	1.66 ± 0.69	p < 0.0001*
Gingival index	2.44 ± 0.53	0.53 ± 1.00	1.91 ± 1.13	p < 0.001*
Probing depth (mm)	7.56 ± 1.13	3.22 ± 0.44	4.34 ± 1.21	p < 0.0001*
Clinical attachment level (mm)	7.22 ± 1.56	3.56 ± 1.01	3.66 ± 1.86	p < 0.0001*
Gingival margin (mm)	-0.33 ± 1.50	0.33 ± 1.00	0.66 ± 1.80	p = 0.29
Tooth mobility	0.67 ± 0.71	0.22 ± 0.44	0.45 ± 0.84	p = 0.13
*Denotes statistically significant difference between the two visits (p < 0.05).				

### One-walled Periodontal Defects

**Fig 1:**
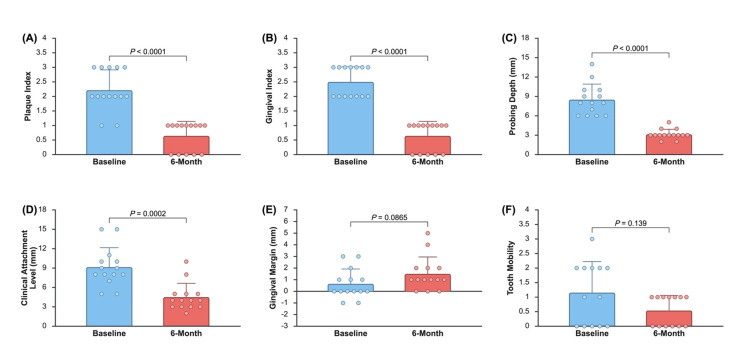
Mean values of periodontal (a) plaque index; (b) gingival index; (c) probing depth; (d) clinical attachment level; (e) gingival margin; and (f) tooth mobility in one-walled periodontal defects assessed at the baseline and six-month follow-up. A p-value < 0.05 indicates a statistically significant difference between the two time points.

From baseline to the 6-month follow-up, statistically significant improvements were observed in the mean values of PI (mean difference [MD]: 1.57 ± 0.86; p < 0.0001) (Fig 1a), PD (MD: 5.36 ± 2.53 mm; p < 0.0001) (Fig 1c), and CAL (MD: 4.35 ± 3.71 mm; p = 0.0002) (Fig 1d, Table 2). However, statistically non-significant outcomes were found in the mean values of GI (MD: 0.86 ± 1.68; p = 0.075) (Fig 1b), GM (MD: 1.00 ± 2.05 mm; p = 0.08) (Fig 1e), and tooth mobility (MD: 0.64±1.15; p = 0.05) (Fig 1f, Table 2).

### Two-walled Periodontal Defects 

**Fig 2:**
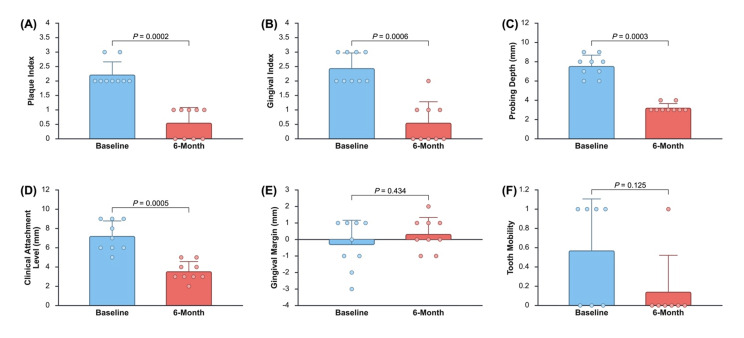
Mean values of periodontal (a) plaque index; (b) gingival index; (c) probing depth; (d) clinical attachment level; (e) gingival margin; and (f) tooth mobility in two-walled periodontal defects assessed at the baseline and the 6-month follow-up. A p-value < 0.05 indicates a statistically significant difference between the two time points.

From baseline to the 6-month follow-up, statistically significant improvements were observed in the mean values of PI (MD: 1.66 ± 0.69; p < 0.0001) (Fig 2a), GI (MD: 1.91 ± 1.13 mm; p = 0.00011) (Fig 2b), PD (MD: 4.34 ± 1.21 mm; p < 0.0001) (Fig 2c), and CAL (MD: 3.66 ± 1.86 mm; p < 0.0001) (Fig 2d, Table 2). However, statistically non-significant outcomes were found in the mean values of GM MD: 0.66 ± 1.80 mm; p = 0.29) (Fig 2e) and tooth mobility (MD: 0.45 ± 0.84; p = 0.13) (Fig 2f, Table 2). At baseline, all patients exhibited positive BOP; however, at the 6-month follow-up, most of the patients showed an improvement, with a shift from positive to negative BOP status. Only 1 patient retained a positive BOP at the 6-month follow-up.

### Frequency Distribution of Residual PD and CAL Gain

At the 6-month follow-up, most of the treated sites showed residual PD < 5mm (n = 24/25; 96%), whereas only 1 site (4%) demonstrated residual PD ≥ 5 mm. Regarding CAL gain, most sites (n = 23/25; 92%) demonstrated a CAL gain ≥ 3 mm, while only 2 sites (8%) exhibited a CAL gain < 3 mm (Table 3).

**Table 3:** Frequency distribution of residual probing depth and clinical attachment level gain at 6-month follow-up

Periodontal parameter	Classification	Number of sites (n)	Percentage (%)
Residual PD (6-month)	< 5 mm ≥ 5 mm	24 1	96% 4%
CAL gain	≥ 3 mm < 3 mm	23 2	92% 8%


## DISCUSSION 

Exosomes are well-established in tissue regeneration through various mechanisms: (a) they facilitate angiogenesis, which is critical for the process of tissue healing;^49^ (b) they modulate the immune response to foster a pro-regenerative environment;^21^ and (c) they deliver various microRNAs that promote tissue formation.^43^ ABBC (Anorganic Bovine Bone Mineral) scaffolds furnish a calcium-rich environment that is conducive to bone mineralization and provides structural support that exhibits osteoconductive properties.^43^ Hence, this clinical study represents a pioneering effort in using exosomes for the treatment of periodontitis in-vivo. Specifically, this study aimed to evaluate the safety of exosomes fabricated for periodontal regeneration (Periosomes) with a collagen-containing bone grafting material (ABBC) and H-PRF for the treatment of advanced periodontal bone loss, with periodontal clinical parameters being assessed at baseline and the 6-month follow-up. It is important to note that all teeth treated in this case series ranged from poor to hopeless, with no single tooth treated given a prognosis above poor according to the McGuire and Nunn’s classification system.^24^

The findings showed that the use of exosomes combined with ABBC scaffolds and H-PRF resulted in statistically significant reductions in periodontal PD and improvements in CAL for both one-walled and two-walled defects from baseline to the 6-month follow-up. Moreover, BOP improved statistically significantly, with almost all patients displaying no BOP after six months. In our study, two-walled defects demonstrated a 4.34 ± 1.21 mm reduction in PD and a 3.66 ± 1.86 mm reduction in CAL, while one-walled defects demonstrated a 5.36 ± 2.53 mm reduction in PD and a 4.35 ± 3.71 mm reduction in CAL at 6 months. The greater improvement observed in one-walled defects may be attributed to their more severe baseline CAL and PD, reflecting the regenerative potential of this novel therapy. Moreover, frequency distribution analysis demonstrated that 96% of the sites attained residual PD < 5 mm and 92% of sites demonstrated CAL gains ≥ 3 mm, facilitating the clinical predictability and relevance of the observed findings.

These findings are comparable to a study by Sculean et al^40^ in which Bio-Oss Collagen, with a resorbable collagen membrane, was compared to open flap debridement. In the ABBMC group, they reported average reductions in PD and CAL of 5.4 ± 0.9 mm and 4.1 ± 0.9 mm, respectively, at the 1-year follow-up. While their outcomes were slightly higher in comparison, their baseline PD and CAL were also higher, and the majority of their treated defects were two-walled, which are generally more favorable for regenerative outcomes.^40^ Additionally, they reported 1.3 ± 1.0 mm recession, while in our study, only 1.00 ± 2.05 mm and 0.66 ± 1.80 mm recession were observed in one-walled and two-walled defects, respectively.^40^ In a similar study, Nevins et al^34^ evaluated four cases utilizing Bio-Oss Collagen with a resorbable collagen membrane and reported an average improvement of 5.75 mm in PD and 5.25 mm in CAL. However, they also reported higher baseline recordings, and their sample size was limited to just four cases.^34^ A split-mouth study by Sezgin et al^41^ involving 15 patients, the use of ABBM + PRF was compared to ABBM alone and found that the PRF group showed statistically significantly greater CAL improvements at six months with an average CAL reduction of 4.47 ± 1.60 mm, similar to our findings. Nonetheless, direct comparison to these studies is limited due to the differences in the intervention, as none of the aforementioned studies utilized exosomes and/or also employed a collagen membrane instead of H-PRF. Unfortunately, no study to date has investigated the combination of solely utilizing ABBMC (Anorganic Bovine Bone Mineral Collagen) and H-PRF exclusively, presenting a relevant gap in the literature. Overall, the similarity in outcomes supports the regenerative effectiveness of ABBM yet underscores the need for future randomized clinical trials isolating exosomes as a variable in periodontal regeneration. The primary aim of this study was to test for the first time in human periodontitis patients the safety and feasibility of utilizing exosomes.

Based on pre-clinical studies, the combination of exosomes with a bone-derived scaffold has been shown to promote both bone and periodontal regeneration, thereby facilitating the regeneration of both one-walled and two-walled periodontal defects.^17,37^ The utilization of PRF as a tissue engineering scaffold has been demonstrated in numerous studies as an effective delivery of biomolecules such as exosomes, while the ABBMC was utilized due to space maintenance.^48^ The combined use of both ABBC and H-PRF functions to hold and deliver the exosomes, potentially enabling the sustained release of the bioactive factors over an extended period.^48^ This prolonged effect might have significantly contributed to the marked improvements documented at the six-month follow-up. The observed reduction in PI values indicates improved oral hygiene, which may be attributable to the increased ease of maintenance at the treated sites. The reductions in PD and CAL serve as direct indicators of improved periodontal health and tissue regeneration.

The statistically significant improvement observed in periodontal GI from baseline to the 6-month follow-up pertaining specifically to two-walled defects implies that the combination of exosomes and ABBC scaffolds was particularly efficacious in mitigating inflammation with this category of defect. This might be associated with (a) improved retention of the biomaterials within two-walled defects, which facilitates an extended release and functionality of exosomes; and (b) superior vascularization and cellular infiltration in two-walled defects in comparison to one-walled defects, thereby promoting the regenerative capabilities of the exosomes. The presence of statistically non-significant GI improvement in one-walled defects might be attributed to (a) the diminished stability of the scaffold within one-walled defects, which could result in suboptimal exosome delivery; and (b) the increased complexity in the regeneration of one-walled periodontal defects owing to the scarcity of surrounding healthy tissue.

Exosomes have demonstrated pronounced anti-inflammatory features in many previous studies.^9^ In the context of this study (i.e., periodontitis), Periosomes were hypothesized to modulate the local immune response, thereby reducing excessive inflammation. Moreover, exosomes regulate pro-inflammatory cytokine synthesis, fostering a more equilibrated inflammatory environment.^8^ Notably, future in-vitro and in-vivo research specific to the clinically available Periosomes is needed to better understand the mechanisms by which they impact periodontal tissue healing in humans.

### Study Strengths

This study dives into a novel therapeutic modality for periodontitis through the use of exosomes, the smallest subset of extracellular vesicles that play pivotal roles in intercellular communications.^35^ This strategy signifies a paradigm shift from conventional periodontal therapies towards a more precise, acellular treatment approach. Traditional periodontal therapies are frequently limited in terms of achieving comprehensive tissue regeneration and sustained stability.^50^ Through the combination of exosomes with a scaffold, this research aimed to improve periodontal regeneration and potentially overcome some of the previous limitations found in routine clinical practice. Hence, this investigation serves to bridge the divide between pre-clinical research and clinical practice. Executing research in a real-world clinical setting provides invaluable insights regarding the feasibility and safety of exosome-mediated therapies for periodontitis in humans.

As mentioned previously, exosomes are now available clinically and have been utilized in thousands of patients in medical clinical studies.^26^ To date, however, only a single case report by our group has investigated their use in regenerative dentistry,^12^ despite over 100 pre-clinical publications on the topic.^22^ The present clinical study seeks to substantiate these advantages in the treatment of advanced periodontal bone defects, potentially resulting in enhanced clinical outcomes in comparison to existing standard treatment modalities. By evaluating clinical parameters at baseline and 6 months postoperatively, this initial pilot study offers a comprehensive assessment of the combined treatment’s efficacy. Should this strategy prove successful in future randomized clinical studies when compared to standard biomaterials, it could lay the groundwork for the development of novel therapeutic strategies in periodontal regeneration as well as other dental and oral health applications.

### Limitations and Future Recommendations

The limitations of the study are important to recognize. First, with only 13 patients and 23 intrabony defects, the study’s small sample size may not accurately represent the broader population affected by periodontitis. Additionally, the sample included primarily non-smokers with good systemic health, limiting applicability to other patient demographics. Future studies could expand the sample size and diversify patient demographics. Including a broader range of patient health states, for example, those with controlled or uncontrolled systemic conditions such as diabetics, could yield more comprehensive and generalizable data. Second, the six-month follow-up duration might be inadequate to observe long-term effects, particularly for parameters including GM positioning and tooth mobility, which often require extended observation to detect significant changes. A longer follow-up period (12 to 24 months) could provide more conclusive insights into the stability and sustainability of the regenerative effects. Third, all treatments were performed by a single clinician, which could introduce bias and limit the generalizability of the outcomes. Incorporating multiple clinicians in future studies could help take operator variability into account and increase the study’s reliability. Another important limitation is the lack of a control group using conventional treatments (such as ABBC scaffolds/H-PRF without the use of exosomes), which restricts the study’s ability to assess the precise efficacy of the exosomes. Thus, which of the regenerative capacities of this treatment modality were from the scaffold, H-PRF, or exosomes remains to be determined. Moreover, supportive periodontal therapy during the follow-up period may influence clinical outcomes, especially PI and GI. This adjunctive therapy may confound the treatment effects specifically attributable to exosome therapy. Additionally, differences in defect morphology (i.e., one-walled vs two-walled defects) may affect treatment outcomes and the interpretation of results. Stratifying the sample by defect morphology could lead to more precise insights into how defect characteristics influence outcomes. Finally, separating patients into groups based on various regenerative approaches in randomized clinical trials could clarify whether clinical improvements are due to exosome therapy alone or other commonly utilized biomaterials.

Therefore, the present findings should be interpreted with caution as the combined approach lacking a control group considerably limits the ability to isolate the efficacy of exosomes in periodontal regeneration. The aim of this pilot case series was to demonstrate the safety and feasibility of utilizing exosomes in combination with H-PRF and ABBMC in periodontal regeneration. Future randomized controlled trials should include comparator groups in order to individually evaluate the efficacy of exosomes with longer follow-up radiographic and defect standardization, and patient-reported outcomes.

## CONCLUSION 

To our knowledge, this is the first human clinical study investigating the safety and feasibility of exosomes in periodontal regenerative therapy. This pilot case series demonstrated that the use of exosomes when combined with ABBMC/H-PRF was a safe and feasible treatment modality for advanced periodontal osseous defects. The findings highlight the role of defect morphology in therapeutic efficacy and suggest that exosome-scaffold combinations could serve as a targeted treatment option for periodontal regeneration.

## ACKNOWLEDGEMENT

Nima Farshidfar is a recipient of the 2024 Research Scholarship from Osteology Foundation.

## CONFLICT OF INTEREST 

Richard J. Miron holds intellectual property on the production of PRF. All other authors declare that they have no conflict of interest.

**Fig 3:**
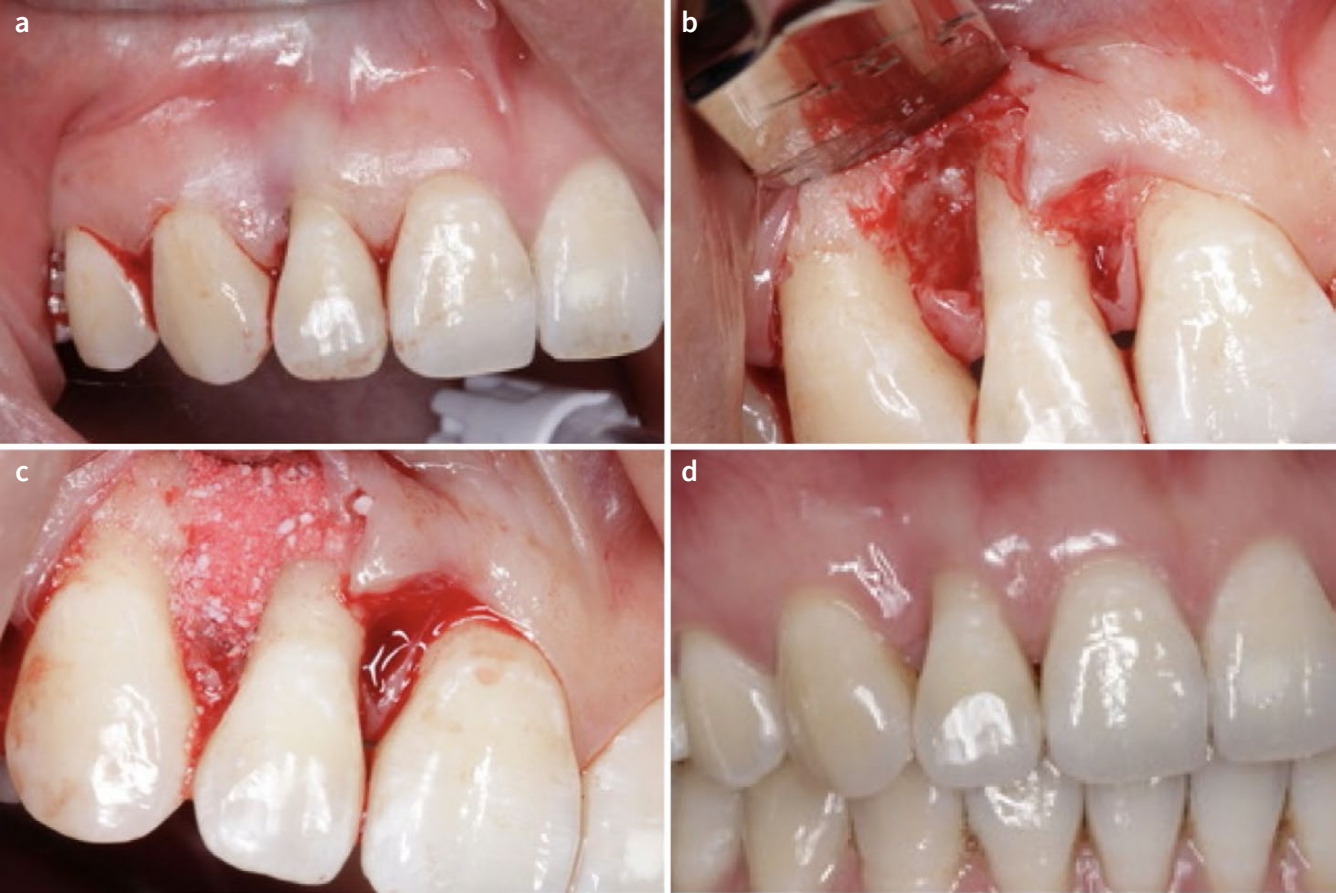
Clinical photos of periodontal surgery. a) Pre-operative view of tooth no. 12 with a severe intrabony defect. b) Full-thickness flap reflection revealing the intrabony defect, which was degranulated utilizing curettes and a carbon dioxide laser. c) Application of the graft complex containing Periosomes, ABBC, and H-PRF. d) 6-month post-operative photograph demonstrating absence of inflammation.

**Fig 5:**
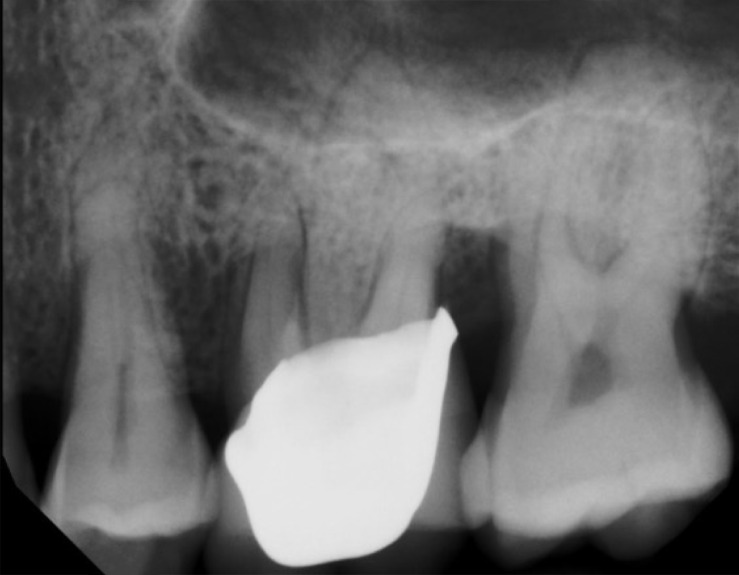

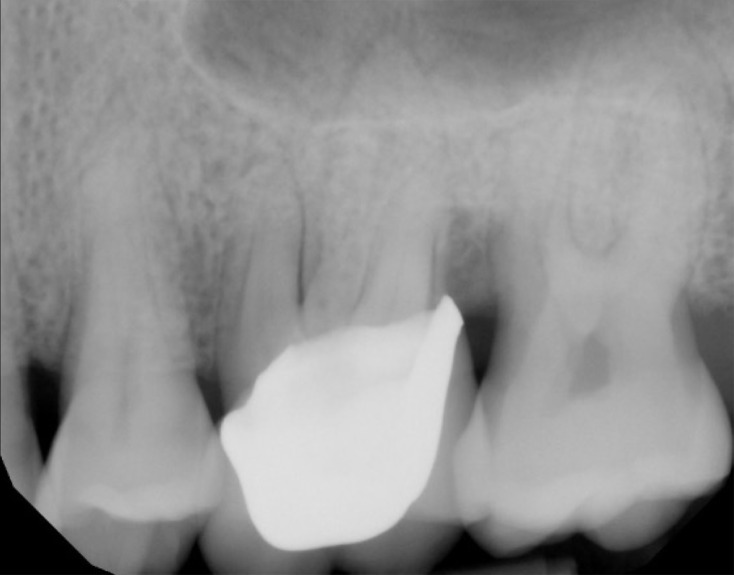
Pre-operative and 6-month post-surgery radiographs demonstrating significant bone fill at sites 25 to 27.

**Fig 4:**
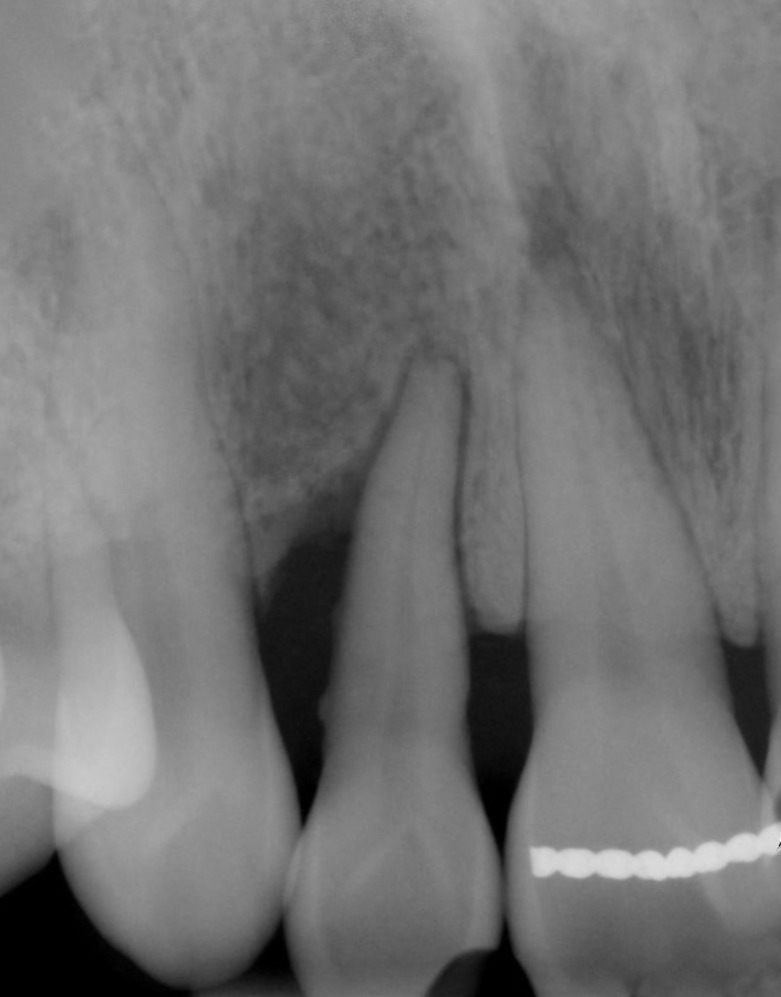

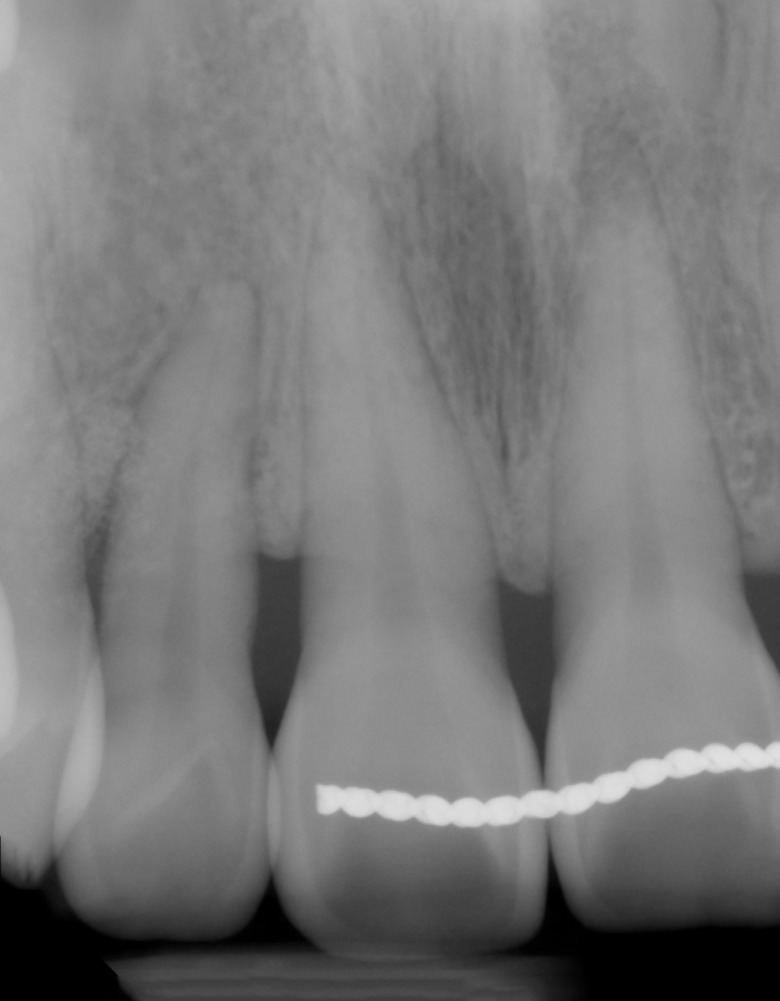
Pre-operaive and 6-month post-surgery radiographs demonstrating significant bone fill at site (tooth no. 12) in the same case as Fig 3.
